# Hierarchy and hope: Exploring AI’s role in medicine through a thematic analysis of online discourse

**DOI:** 10.1371/journal.pdig.0001212

**Published:** 2026-01-30

**Authors:** Johan Pushani, Sherwin Rajkumar, Alishya Burrell, Erin Peebles, Amrit Kirpalani

**Affiliations:** 1 Department of Paediatrics, Schulich School of Medicine and Dentistry, Western University, London, Ontario, Canada; 2 Department of Medicine, Schulich School of Medicine and Dentistry, Western University, London, Ontario, Canada; 3 Department of Paediatrics, Faculty of Medicine, University of British Columbia, Vancouver, British Columbia, Canada; 4 Division of Nephrology, Children’s Hospital, London Health Sciences Centre, London, Ontario, Canada; Shahid Beheshti University of Medical Sciences School of Dentistry, IRAN, ISLAMIC REPUBLIC OF

## Abstract

The healthcare community remains divided on the benefits of artificial intelligence (AI) in medicine. In this qualitative study, we sought to better understand the perceived opportunities and threats of AI among premedical students, medical students, and physicians. We conducted a thematic analysis on Reddit, a social platform where candid opinions are often shared. Posts from the r/premed, r/medicalschool, and r/medicine subreddits were searched using the terms “AI”, “chatGPT”, “openAI”, and “artificial intelligence”. We analyzed 2403 comments across 47 threads from December 2022 to August 2023. A coding scheme was developed manually following Braun and Clarke’s (2006) framework, and common themes were extracted. The main themes identified centered on AI enhancement versus replacement. Careers perceived to be lower in the medical social hierarchy were considered most at risk of replacement. AI was thought to first replace non-medical jobs, followed by mid-levels, and then primary care and diagnostic specialties, with specialists and surgeons affected last. Some contributors emphasized that AI could never replace a physician’s compassion and nuanced clinical judgment. Others viewed AI as a tool to enhance efficiency, particularly in tasks such as studying, note writing, screening, and triage. Although verifying the credentials of commenters on online forums poses a challenge, platforms like Reddit offer a valuable opportunity to understand nuanced attitudes and perceptions regarding AI in medicine. Online forums allow for a unique understanding of the impressions of AI in medicine. While AI was generally well-received, we identified a key finding: a socially hierarchical, biased form of thinking among healthcare professionals. The perpetuation of this biased mindset may contribute to role devaluation, mistrust, and collaboration challenges within healthcare teams–ultimately impacting patient care. To fully leverage AI’s potential in medicine, it is critical to acknowledge and address potentially biased perceptions within the healthcare community.

## Author summary

Artificial intelligence (AI) tools, like ChatGPT, are rapidly becoming part of healthcare, yet there is still uncertainty about whether AI will primarily *support* clinicians or *replace* them. In this study, we analyzed public online discussions to better understand how these groups talk about AI in medicine. Using thematic analysis, we reviewed 2,403 comments across 47 Reddit threads from December 2022 to August 2023. We found two dominant themes. First, many users viewed AI as an *enhancement tool*, supporting studying, writing, clinical documentation, and early screening or triage - while emphasizing that AI can be inaccurate and requires human oversight. Second, others emphasized AI as a potential *replacement force*, often predicting a “hierarchy” of job risk where roles perceived as lower in the medical social structure were viewed as more vulnerable to automation than specialist physicians. These findings suggest that opinions about AI are shaped not only by technology, but also by professional identity and hierarchy, factors that may influence collaboration and the equitable implementation of AI in healthcare.

## Introduction

The integration of artificial intelligence (AI) in healthcare marks a pivotal juncture, with AI’s potential to revolutionize diagnostics, treatment, and patient care at the forefront of contemporary medical discourse. Yet, amidst this technological advance, the medical community’s reception remains mixed–characterized by both optimism for AI’s transformative capabilities and apprehensions regarding its impact on professional roles and patient care [1–3].

The need to investigate these perceptions is underscored by the rapid evolution of AI technologies and their implementation in healthcare environments. As innovation outpaces policy and practice, aligning technological advancement with healthcare delivery and professional norms becomes increasingly urgent [4]. To support this alignment, a deeper understanding of healthcare professionals’ perceptions of AI is needed.

While previous survey-based studies have explored medical professionals’ perceptions of AI, spontaneous and candid perspectives remain underexamined. Our qualitative study analyzed Reddit discussions to capture unfiltered views on the opportunities and challenges AI is perceived to bring to the medical field. By incorporating the perspectives of premedical students, medical students, and physicians, we provide a broader snapshot of how AI is perceived across the current and future medical workforce.

Although the credentials of online commenters cannot be verified and these comments do not represent the medical field as a whole, open discussion on platforms like Reddit offer valuable insight into nuanced and candid opinions surrounding AI in medicine. These conversations can reveal not only individual opinions but also implicit attitudes and underlying structural themes–such as role prestige and the social hierarchy within medicine– that shape how different groups discuss and engage with AI. This work complements traditional survey-based studies and is beneficial in guiding the ethical development of AI solutions while safeguarding the integrity of medical practice and patient care amidst this digital transformation [5].

## Methods

In this qualitative study, we analyzed discussions on the topic of AI in medicine on the social media site Reddit. Our primary goal was to explore and understand perceptions around AI in medicine amongst pre-medical students, medical students, and physicians.

### Platform

Reddit is a popular social news aggregation and discussion platform that is widely used by students and physicians. Users can ask questions, disseminate information and publicly share experiences. Submitted content, or threads, are displayed in their appropriate categories, or subreddits, for all users to interact. As of October 2023, there are over 100 thousand active communities and 16 billion posts and comments [6]. The specific subreddits analyzed in the paper include r/premed with 388 thousand members, r/medicalschool with 715 thousand members, and r/medicine with 461 thousand members.

While Reddit users typically post under pseudonyms, making individual identities and credentials unverifiable, the platform remains a credible data source for qualitative research. Although anonymity limits demographic precision, it also facilitates spontaneous, unfiltered discourse—offering insight into genuine attitudes and experiences, particularly within Reddit’s topic-specific communities organized around shared interests [7]. Anonymity further reduces social desirability bias, enabling users to express perspectives they might withhold in identifiable settings. This allows researchers to capture authentic concerns rather than performative responses, and encourages discussion of sensitive or controversial professional topics that are rarely addressed in formal surveys. Subreddit-specific moderation policies, community voting systems, and participation norms further support content quality and relevance [8]. Reddit has also been increasingly adopted in academic studies exploring health-related topics due to its large, active user base and transparent, publicly accessible data [9]. These characteristics make it a valuable platform for thematic and exploratory qualitative analysis.

### Theoretical framework

Thematic analysis offers a flexible and rigorous approach for examining qualitative data, particularly suited for analyzing the perceptions and attitudes of diverse groups regarding artificial intelligence (AI) in healthcare [10]. This method allows for the identification, analysis, and reporting of patterns within data, providing a comprehensive and detailed understanding of the discussions around AI in medicine.

For this study, we adopted a contextualist approach, which acknowledges the ways individuals create meaning from their experiences within broader social contexts [10]. This perspective is essential for capturing the complex views on AI among different groups within the medical community.

### Data collection

We searched the r/premed, r/medicalschool, and r/medicine subreddits for all discussion threads created between December 2022 and August 2023, using the terms “AI”, “chatGPT”, “openAI”, and “artificial intelligence”. The content of each thread was reviewed by two researchers (JP and SR) and manually coded to enhance contextual understanding and researcher reflexivity. The initial phase involved immersing ourselves in the data through repeated readings of Reddit threads to fully understand the content. Threads relating to the search terms but not providing opinions or stances were excluded from coding, along with threads labelled as “Meme” and “Shitpost”.

Inductive coding was performed on each data set by two researchers (JP and SR) who independently reviewed threads and generated a set of preliminary codes. The code sets from each thread were compared between researchers and assessed for similarities and differences. If a thread was coded differently by each researcher, a discussion was had regarding reasons for selecting a certain code. Eventually, one of the two codes were selected or an overarching code was used as a middle ground. These code sets were compared and after finding similarities in coding, researchers coded the remaining threads together. The coding process was cyclical, wherein researchers returned to the data after an initial pass at coding, as well as dynamic, wherein codes were refined and grouped to highlight similarities and minimize redundancy.

A thematic analysis was initially performed by three researchers (JP, SR, AK) during a group discussion and meeting. Themes were identified by grouping related codes and examining overarching patterns and their interactions [10]. We then used triangulation by involving two additional researchers (AB, ERP), who were not part of the original data collection, to provide additional perspective and refine the analysis. Thematic saturation was established by iteratively analyzing data until no new themes or insights emerged, demonstrating redundancy in findings. This process ensures a thorough representation of the phenomenon under study. Despite the absence of inter-rater reliability statistics, the limitation was mitigated through triangulation and reflexivity.

### Reflexivity

The researchers in this study had an interest in medical technologies such as AI and share a common belief that AI will make an impact in the medical field, albeit with no stance on the directionality or extent of its implication. We reflected individually and as a group on our potential biases and their impact on our data analysis. Naturally, as medical students and physicians, our role and socialization within the medical education system may have shaped our interpretations. We acknowledge that our experiences may impact the interpretation of the data.

### Ethical considerations

As per University protocol, this study did not require any ethics board approval as it was purely a thematic analysis of discourses from a website with public access. No contact was made with users and there was no risk or harm to any individual. Our team functioned exclusively as observers.

## Results

We analyzed a total of 2403 comments across 47 threads from December 2022 to August 2023 (Table 1). Seventeen threads containing 832 comments were coded from r/medicalschool. Eighteen threads containing 1319 comments were coded from r/medicine. Twelve threads containing 252 comments were coded from r/premed.

**Table 1:** AI-related search terms and number of relevant threads analyzed from medical subreddits.

Search Term	Subreddit	Number of Threads Found	Number of Threads Included
**ChatGPT**	r/medicalschool	47	10
	r/premed	47	8
	r/medicine	14	10
**openAI**	r/medicalschool	10	1
	r/premed	3	0
	r/medicine	5	1
**AI**	r/medicalschool	94	4
	r/premed	52	4
	r/medicine	30	6
Artificial Intelligence	r/medicalschool	6	2
	r/premed	4	0
	r/medicine	2	1

**Fig 1:**
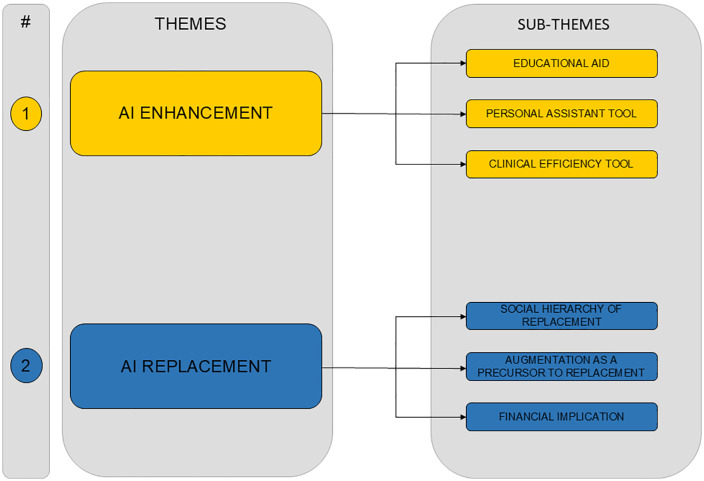
Flowchart of main themes and subthemes.

Our analysis revealed two principal themes: AI Enhancement, highlighting AI’s capacity to bolster medical practices through educational support, writing assistance, and operational efficiencies; and AI Replacement, exploring the contentious debate over AI’s potential to supplant human jobs within medicine (Fig 1). Through the lens of our thematic analysis, we uncovered the multifaceted views of the medical community, reflecting a balance of optimism and concern regarding AI’s evolving role in healthcare.

Whereas much of the discussion around generative AI used ChatGPT as an example, the discourse largely used this tool as a jumping off point for larger level discussion around AI on a grander scale.

### Theme 1: AI enhancement

The first major theme was AI enhancement, where many users framed AI as more than a facilitative tool but as a pivotal element in advancing medical education, writing, and predictive healthcare management. Discussions centered around AI’s multifaceted benefits, while acknowledging its limitations, and scrutinizing the implications of these technological interactions in healthcare.

#### Educational aid.

The role of AI as a dynamic educational resource was evident, with students leveraging its capabilities to generate study materials, including licensing exam-style questions and creative mnemonics. This innovative use of AI facilitates a deeper understanding and retention of information, as one participant noted, “I asked it to generate NBME [National Board of Medical Examiners]-style questions... It’s useful as a quick reference and especially useful as a way to re-organize information... generate mnemonics etc.” (Thread 8, Table 2). The sentiment was echoed by another, who found AI “beneficial for summarising or directing your study... asking for mnemonics or poems or references to study from” (Thread 8, Table 2). These insights underscore AI’s potential to simplify complex concepts, making educational content more accessible, as illustrated by the comment, “I use it to help me study ‘explain X and Y like I’m 5 with examples’... It will only get smarter” (Thread 25, Table 2).

**Table 2:** Summary of Reddit threads identified by search terms and their corresponding number of comments.

Search Term	#	Thread Name	Subreddit	# of Comments
**ChatGPT**	1	Using AI/ChatGPT to help with research paper	r/medicalschool	19
	2	Anyone been using Chat GPT as a superior google search for studying	r/medicalschool	4
	3	With the rise of AI which medical specialties will be the first to fall?	r/medicalschool	34
	4	I’m not 100% I picked the right career after witnessing the rise of AI	r/medicalschool	21
	5	A Twitter user claims GPT-4 saved his dog’s life after a vet couldn’t correctly diagnose her symptoms	r/medicalschool	29
	6	Are any medical students using ChatGPT for learning? What prompts are you using?	r/medicalschool	8
	7	ChatGPT	r/medicalschool	9
	8	ChatGPT usage in studying	r/medicalschool	19
	9	ChatGPT passes USMLE	r/medicalschool	79
	10	Pre-Print Study: ChatGPT Approaches or Exceeds USMLE Passing Threshold	r/medicalschool	93
	11	ChatGPT Fails the Multiple-Choice American College of Official journal of the American College of Gastroenterology | ACG	r/medicine	61
	12	ChatGPT has better bedside manner than doctors, study finds	r/medicine	16
	13	Anyone brave enough to use ChatGPT for patient letters?	r/medicine	67
	14	[serious] ChatGPT now passes USMLE, yes that USMLE, with flying colours. Tell me why majority of doctors won’t be replaced by AI in the coming decade.	r/medicine	46
	15	Chat GP passes medical license exam.	r/medicine	132
	16	ChatGPT in medicine	r/medicine	204
	17	In what ways have you integrated ChatGPT in your work, and has it improved your workflow?	r/medicine	92
	18	Performance of ChatGPT on USMLE: Potential for AI-assisted medical education using large language models | PLOS Digital Health	r/medicine	14
	19	ChatGPT Vs USMLE	r/medicine	43
	20	ChatGPT manages Acute MI	r/medicine	55
	21	Medicine replaced by AI?	r/premed	10
	22	How does one “develop writing skills” as a premed?	r/premed	40
	23	What are y’all’s opinion on using CHATGPT for applications?	r/premed	24
	24	Rate ChatGPT’s Personal Statement for Harvard Medical School	r/premed	57
	25	How y’all feeling about ChatGPT?	r/premed	10
	26	I trained chat gpt to grade writing for personal statements.	r/premed	15
	27	Hot take: ChatGPT can be a really useful tool in writing secondaries because it’s a good way to check that you’re not being too robotic	r/premed	1
	28	Why you shouldn’t use ChatGPT to write an essay	r/premed	56
**openAI**	29	Which medical specialties have the least job security?	r/medicalschool	167
	30	OpenAI manages a GI bleed	r/medicine	184
**AI**	31	Is Radiology really a dying specialty?	r/medicalschool	64
	32	Why do some people think AI will replace radiologists?	r/medicalschool	21
	33	Is AI a threat to radiology?	r/medicalschool	49
	34	Will AI take over Radiology? Is it futile to go for Radiology residency now?	r/medicalschool	90
**AI**	35	Can you imagine AI powered EMRs?	r/medicine	101
	36	Medicine and AI- my thoughts	r/medicine	136
	37	What is the future of AI in healthcare?	r/medicine	16
	38	Anyone using AI for learning?	r/medicine	24
	39	Is AI coming for us all?	r/medicine	54
	40	How will machine learning affect medicine?	r/medicine	41
	41	AWS this week unveiled a generative AI scribe for docs. The competition is fierce.	r/premed	3
	42	Medicine replaced by AI?	r/premed	10
	43	Is middle level encroachment and the potential of AI enough of a reason to not start the medical journey?	r/premed	6
	44	What will the role of doctors become when AI takes over?	r/premed	20
**Artificial Intelligence**	45	Which specialties will be most impacted by reduced demand for physicians because of artificial intelligence?	r/medicalschool	8
	46	Yesterday I saw my first ai ct read in a patient’s chart	r/medicalschool	118
	47	Comparing Physician and Artificial Intelligence Chatbot Responses to Patient Questions Posted to a Public Social Media Forum	r/medicine	33

However, concerns were raised about AI’s reliability, with numerous comments highlighting the tendency of ChatGPT to provide incorrect or misleading information, or as one user deemed “something between incorrect answers presented with made-up citations… to answers that are just slightly off” (Thread 6, Table 2). The collective viewpoint suggests a cautious approach: “it can be wrong at times, so you gotta use your best judgement” (Thread 8, Table 2), emphasizing the need for critical engagement with AI-generated content.

#### Personal assistant tool.

The utility of AI as a writing tool was another main point of discussion. Users overwhelmingly found that ChatGPT was helpful when writing essays and literary works, and appreciated that this would be particularly valuable in medicine, where the clarity and precision of communication are paramount. Users appreciated AI for “having writer’s block? Let chatgpt [sic] give you a starting point... Too tired to edit an essay? Ask chatgpt [sic] to find grammar and spelling mistakes” (Thread 25, Table 2).

Appreciation was also given to the potential for AI to reduce the time spent on administrative tasks that were felt to be mundane or uninteresting:

“My attending wrote some insurance appeal letters with it after we were joking about chatGPT [sic] use. He was impressed with the result.”

Yet, the absence of personal touch in AI-generated content was noted, highlighting a trade-off between efficiency and the nuanced expression of human emotions and stylistic preferences. Many users agreed that generative AI “removes some of the pathos and stylistic choices” (Thread 22, Table 2) or that “the main lacking trait was a personal touch or personal voice” (Thread 26, Table 2), however many were quick to emphasize that “you can always edit the draft chatgpt [sic] makes” (Thread 22, Table 2) suggesting this may not be as significant a limitation.

#### Clinical efficiency tool.

Another helpful area for AI is its triaging ability and predictive capacity. Some users believed AI could leverage these abilities to increase efficiency and better evaluate outcomes and potential complications based on certain patient features.

The most likely answer is that machine learning may increase our efficiency and accuracy by processing large quantities of data and automating repetitive tasks with its output being “preliminary” and requiring the review and editing of a physician (think writing notes, triage, oxygen weaning recommendations, maybe even prelim radiology reads or at least highlighting areas of likely interest). (Thread 4, Table 2)

While it shows promise in these areas, users agreed that it still lacks refinement and overall reliability. As such, its current role is that of a screening tool rather than a diagnostic one.

“There is a fairly common AI service that most hospitals have been using for years that will screen CT heads for bleeds, CTAs for large vessel occlusions, and CT PE studies for PE. It is OK but quite frequently misses clinically relevant findings, and more or less doesn’t look for any pathology outside the listed diagnoses.” (Thread 46, Table 2)

### Theme 2: AI replacement

Many discussions framed AI as more than an assistive tool, but rather, one with the potential to make specific aspects of human physicianship obsolete. The discourse on AI’s potential to replace jobs in medicine uncovered a complex narrative that delves into the social hierarchy of replacement, conditional acceptance of AI’s role, and the nuanced implications of augmentative technologies. This theme highlights the medical community’s divided stance on AI, reflecting a spectrum of opinions that range from cautious optimism to outright skepticism, all framed within the broader context of healthcare’s evolving landscape.

#### Social hierarchy of replacement.

At the heart of the discussion was the concern over AI’s capability to replace human roles within medicine, sparking a debate that touches on the fundamental aspects of medical practice. The conversation was anchored in a perceived hierarchy of vulnerability to AI replacement with users suggesting a tiered approach to AI’s integration, where non-medical jobs were seen as the most vulnerable, followed by “mid-level healthcare workers” (allied health professionals), and with physicians, particularly subspecialists, viewed as less at-risk. One participant starkly noted, “By the time AI is able to replace doctors, they’ll have already replaced basically every other knowledge worker” (Thread 9, Table 2), implying a gradual encroachment of AI across professions.

The dialogue evolves to address the medical field specifically, where mid-level practitioners such as NPs and PAs are viewed as more immediately susceptible to AI’s influence than physicians, a sentiment captured by the assertion that “an introduction of AI won’t skip straight to the top and replace doctors” (Thread 21, Table 2). This distinction extends within the physician community itself, with primary care practitioners perceived as more vulnerable compared to specialists and surgeons. The latter group is considered the least at risk due to the complex nature of surgical procedures, as highlighted by one user: “We are nowhere remotely close to AI touching surgery” (Thread 29, Table 2).

Contrasting with these views are arguments emphasizing the irreplaceable aspects of human judgment, complex decision-making, and holistic patient care that AI cannot mimic. The skepticism about AI assuming total responsibility is underscored by concerns over legal liabilities, with one participant remarking, “I honestly don’t think AI will ever result in endangering physicians’ jobs” (Thread 3, Table 2), reflecting doubts about AI’s full integration into clinical settings.

Amidst these varied perspectives, some comments employ satire to diminish fears of AI replacement, likening them to historical apprehensions about technological advancements rendering human skills obsolete, “It’s like when calculators were invented and they got rid of all the mathematicians” (Thread 34, Table 2). However, the prevailing sentiment is not one of dichotomy but rather a nuanced view that AI will transform, not replace, medical practice. This view is exemplified by the belief that AI will serve as an augmentative force, enhancing diagnostic support and triaging in primary care, “AI is more likely to complement healthcare professionals rather than replace them entirely” (Thread 29, Table 2).

Through these discussions, a consensus emerged that while AI is set to significantly influence medical practice, it is anticipated to act as an assistive technology, bolstering the efficiency and capability of healthcare delivery without displacing the essential role of human professionals in medicine. This narrative reflects a collective anticipation of AI as a transformative, yet non-displacing, force within healthcare, reinforcing the enduring value of human expertise in the field.

#### Augmentation as a precursor to replacement.

Whereas many users favoured the role of AI as an assistive tool for physicians, many stressed the secondary effect of this in reducing the total number of workers. If alongside AI physicians could increase their productivity, then users anticipated a decrease in the number of physicians needed by a hospital.

“I don’t see AI ever totally causing job obsolescence. However, I can see diagnostic radiology experiencing the first effects in the form of reduction in the number of jobs. Imagine an AI that can screen hundreds of images in minutes and highlight any perceived abnormality, and kicking it over to be reviewed by a human who then verifies the “abnormality” as a pathological issue or normal.” (Thread 3, Table 2).

The anticipation of AI’s role in diagnostic radiology as a precursor to broader impacts across the field reflects a pragmatic view of technology’s integration into healthcare, “If it can allow one person to do the work of 5, it’s game over for 80% of people” (Thread 5, Table 2).

#### Financial implication.

As a result of AI increasing physician efficiency and productivity, many users raised concerns about changes to compensation structures,“your job will likely become a lot faster since you’ll mostly screen for the AI detections to see if they’re false positives/negatives, which means you’ll be expected to do a lot more during your time and get compensated the same.” (Thread 34, Table 2).

A small proportion of users explored compensation changes through the lens of hospital administrators and how hospitals are set to profit more from proper AI implementation. In the eyes of the hospital, “physicians are expensive to employ and ‘wasteful’ (Thread 29, Table 2). Concerns about potential changes to compensation structures, with AI offloading tasks from physicians, hint at the economic dimensions of AI’s adoption. The expectation that hospitals might employ fewer physicians to maintain profitability underscores the potential economic pressures driving AI’s integration, “In any case, [AI] dramatically reduces the need for physicians on staff, and hospitals will not hold onto useless staff members” (Thread 29, Table 2).”

The discourse on AI replacement in medicine was characterized by a recognition of AI’s transformative potential alongside a critical appraisal of its limitations. The nuanced discussions reflected conditional acceptance of AI’s inevitable role, while contrasting perspectives on its impact on the future healthcare workforce.

## Discussion

Our thematic analysis of discourse on AI in medicine revealed a landscape characterized by both enthusiasm for AI’s potential to enhance various aspects of medical practices and apprehension about its capacity to replace human roles. The identified themes of AI Enhancement and AI Replacement capture a collective anticipation of AI’s transformative impact, tempered by concerns about its implications for the medical workforce. This nuanced perspective highlights the complex dynamics of integrating AI into healthcare, reflecting a cautious optimism where AI is perceived both as a complement to and a challenger of traditional medical roles.

Discussions around AI in educational support, writing assistance, and clinical efficiency reflect optimism about its ability to augment healthcare delivery [11,12]. These perspectives align with best practices in the field, supporting AI’s role as a tool that enhances, but does not replace professional expertise [13]. Nevertheless, concerns regarding AI’s limitations also emerged, underscoring the ongoing need for human oversight to ensure safe and reliable AI applications [14].

Discussions also touched on AI’s capacity to replace human jobs within the medical hierarchy, revealing a nuanced debate that extends beyond technological feasibility to touch on social and professional hierarchies. This perspective on AI’s role in healthcare—where the perceived replaceability of human roles by AI is influenced by one’s position within the medical hierarchy—raises important questions about the intersection of technology, power, collaboration, and professional identity.

Our findings suggest that professionals perceive AI’s threat to replace human roles as stratified across different levels of the medical hierarchy. Notably, tasks performed by those lower in the hierarchy are perceived as more vulnerable to automation, while those requiring complex judgment by physicians and specialists are viewed as less replicable. This hierarchy-driven perception highlights how social and professional structures influence expectations about AI’s impact, reflecting broader workforce concerns about technology’s effects on jobs [15]. These views are also consistent with literature indicating that tasks requiring high perceived levels of cognitive complexity and emotional intelligence are less likely to be replaced by AI [16]. Such perspectives emphasize the enduring importance of human qualities in healthcare: empathy, moral judgment, and the physician-patient relationship, which may be difficult to replicate with AI. Emanuel and Wachter further support this view by arguing for AI as an augmentative tool, emphasizing the irreplaceable value of human judgment in clinical decision-making [17].

The implications of these findings extend beyond individual roles to broader healthcare systems. Hierarchical perceptions may reinforce biases, foster mistrust, degrade communication, and heighten workplace tension. Low interdisciplinary rapport has been repeatedly linked to poorer patient outcomes, undermining both patient-physician trust and public confidence in healthcare–effects which are likely amplified by entrenched social hierarchies [18].

Beyond the clinical setting, hierarchical perceptions carry broader professional and societal consequences. Healthcare hierarchies not only shape workplace culture but also influence policy, often privileging higher-status professionals while marginalizing others [19]. Because higher-ranked professionals are perceived as less replaceable, they may discount AI’s relevance to their own roles and underestimate its evolving capabilities in replicating these sophisticated tasks. Given their disproportionate influence on policy and reform, this perception risks diminishing AI’s value and slowing its adoption. In this way, entrenched hierarchies may obscure AI’s potential to enhance care across all levels of practice. As Emanuel and Wachter argue, such misconceptions may hinder the equitable and effective rollout of AI technologies, underscoring the need to reassess how roles are valued and how AI is integrated into healthcare [17].

Similar dynamics appear in curriculum development, where entrenched hierarchies may limit interdisciplinary representation or AI literacy. Without deliberate adaptation, this risks reinforcing outdated professional silos rather than preparing clinicians for collaborative, technology-enhanced practice. Effective curricula must teach not only AI skills but also interprofessional collaboration in contexts where AI performs routine cognitive tasks. Recent frameworks recommend integrating AI literacy across the learning continuum and expanding interprofessional education so learners understand shifting scopes, task allocation, and shared responsibilities when AI is introduced [20,21]. Failure to do so may lead trainees to over-trust technology or undervalue colleagues, ultimately compromising safe and effective AI implementation.

Taken together, these findings highlight the urgent need to reassess hierarchical assumptions and role valuations in healthcare. Acknowledging and addressing biased perceptions through education, policy reform, and equitable integration is essential to ensure that AI serves as a universal augmentative tool, enhancing care across all levels, rather than reinforcing outdated hierarchies.

## Limitations

Our study is not without limitations. As a qualitative analysis, it does not offer quantitative measures of the prevalence or weight of the identified themes; future research could incorporate such methods to enhance generalizability. Although ChatGPT-4.0 was the most advanced version available during the study period, we did not confirm which version was used during coding, which may have influenced the outputs analyzed. Also, the use of Reddit discussions introduces uncertainty around the authenticity and background of commenters; users are not verified as pre-medical students, medical students, or healthcare professionals, and the platform tends to attract more tech-savvy individuals. Therefore, the voices captured may be disproportionately those of internet-active, self-selecting users and earlier adopters of AI, which may in turn under-represent the perspectives of older clinicians, nurses and allied health professionals, as well as those working in settings with limited digital infrastructure. Additionally, comments were not run through an AI and bot content detector, raising the possibility that some posts were partially or fully generated by language models–an added layer of complexity when interpreting user perspectives. Lastly, the rapid evolution of AI technology and its applications in healthcare may outpace the relevance of these findings, emphasizing the need for ongoing research and dialogue. Future studies using survey-based attitude metrics may bring to light changing attitudes regarding AI in medicine as it becomes increasingly incorporated into the healthcare field.

## Conclusion

We demonstrate the perceived opportunities and threats of AI in medicine. We identify a particular social hierarchical model of replacement, whereby professionals perceived AI’s threat to replace human roles as stratified across different levels of the medical hierarchy. Of note, tasks performed by physicians deemed to be lower in the hierarchy are viewed as more susceptible to AI replacement. This line of thinking could hinder the true potential and benefits of AI in the healthcare field, while also threatening its proper adoption and equitable implementation. We need to recognize and address these potentially biased perceptions in the medical field if we are to fully leverage AI to its utmost potential.

## Supporting information

S1 Table Summary of threads included in thematic analysis. (DOCX)
