# Supplementary material for: Hierarchy and hope: Exploring AI’s role in medicine through a thematic analysis of online discourse
Source: PLOS Digit Health. 2026 Jan 30;5(1):e0001212. doi: 10.1371/journal.pdig.0001212 (PMC12858004; doi:10.1371/journal.pdig.0001212)
Supplement: S1 Table — (DOCX) [file pdig.0001212.s001.docx]

**S1 Table.** Summary of threads included in thematic analysis

| **Search Term** | **#** | **Thread Name** | **Subreddit** | **Comments** |
| --- | --- | --- | --- | --- |
| **ChatGPT** | 1 | Using AI/ChatGPT to help with research paper | r/medicalschool | 19 |
|  | 2 | Anyone been using Chat GPT as a superior google search for studying | r/medicalschool | 4 |
|  | 3 | With the rise of AI which medical specialties will be the first to fall? | r/medicalschool | 34 |
|  | 4 | I'm not 100% I picked the right career after witnessing the rise of AI | r/medicalschool | 21 |
|  | 5 | A Twitter user claims GPT-4 saved his dog's life after a vet couldn't correctly diagnose her symptoms | r/medicalschool | 29 |
|  | 6 | Are any medical students using ChatGPT for learning? What prompts are you using? | r/medicalschool | 8 |
|  | 7 | ChatGPT | r/medicalschool | 9 |
|  | 8 | ChatGPT usage in studying | r/medicalschool | 19 |
|  | 9 | ChatGPT passes USMLE | r/medicalschool | 79 |
|  | 10 | Pre-Print Study: ChatGPT Approaches or Exceeds USMLE Passing Threshold | r/medicalschool | 93 |
|  | 11 | ChatGPT Fails the Multiple-Choice American College of Official journal of the American College of Gastroenterology \| ACG | r/medicine | 61 |
|  | 12 | ChatGPT has better bedside manner than doctors, study finds | r/medicine | 16 |
|  | 13 | Anyone brave enough to use ChatGPT for patient letters? | r/medicine | 67 |
|  | 14 | [serious] ChatGPT now passes USMLE, yes that USMLE, with flying colours. Tell me why majority of doctors won't be replaced by AI in the coming decade. | r/medicine | 46 |
|  | 15 | Chat GP passes medical license exam. | r/medicine | 132 |
|  | 16 | ChatGPT in medicine | r/medicine | 204 |
|  | 17 | In what ways have you integrated ChatGPT in your work, and has it improved your workflow? | r/medicine | 92 |
|  | 18 | Performance of ChatGPT on USMLE: Potential for AI-assisted medical education using large language models \| PLOS Digital Health | r/medicine | 14 |
|  | 19 | ChatGPT Vs USMLE | r/medicine | 43 |
|  | 20 | ChatGPT manages Acute MI | r/medicine | 55 |
|  | 21 | Medicine replaced by AI? | r/premed | 10 |
|  | 22 | How does one “develop writing skills” as a premed? | r/premed | 40 |
|  | 23 | What are y’all’s opinion on using CHATGPT for applications? | r/premed | 24 |
|  | 24 | Rate ChatGPT's Personal Statement for Harvard Medical School | r/premed | 57 |
|  | 25 | How y'all feeling about ChatGPT? | r/premed | 10 |
|  | 26 | I trained chat gpt to grade writing for personal statements. | r/premed | 15 |
|  | 27 | Hot take: ChatGPT can be a really useful tool in writing secondaries because it’s a good way to check that you’re not being too robotic | r/premed | 1 |
|  | 28 | Why you shouldn’t use ChatGPT to write an essay | r/premed | 56 |
| **openAI** | 29 | Which medical specialties have the least job security? | r/medicalschool | 167 |
|  | 30 | OpenAI manages a GI bleed | r/medicine | 184 |
| **AI** | 31 | Is Radiology really a dying specialty? | r/medicalschool | 64 |
|  | 32 | Why do some people think AI will replace radiologists? | r/medicalschool | 21 |
|  | 33 | Is AI a threat to radiology? | r/medicalschool | 49 |
|  | 34 | Will AI take over Radiology? Is it futile to go for Radiology residency now? | r/medicalschool | 90 |
|  | 35 | Can you imagine AI powered EMRs? | r/medicine | 101 |
|  | 36 | Medicine and AI- my thoughts | r/medicine | 136 |
|  | 37 | What is the future of AI in healthcare? | r/medicine | 16 |
|  | 38 | Anyone using AI for learning? | r/medicine | 24 |
|  | 39 | Is AI coming for us all? | r/medicine | 54 |
|  | 40 | How will machine learning affect medicine? | r/medicine | 41 |
|  | 41 | AWS this week unveiled a generative AI scribe for docs. The competition is fierce. | r/premed | 3 |
|  | 42 | Medicine replaced by AI? | r/premed | 10 |
|  | 43 | Is middle level encroachment and the potential of AI enough of a reason to not start the medical journey? | r/premed | 6 |
|  | 44 | What will the role of doctors become when AI takes over? | r/premed | 20 |
| **Artificial Intelligence** | 45 | Which specialties will be most impacted by reduced demand for physicians because of artificial intelligence? | r/medicalschool | 8 |
|  | 46 | Yesterday I saw my first ai ct read in a patient’s chart | r/medicalschool | 118 |
|  | 47 | Comparing Physician and Artificial Intelligence Chatbot Responses to Patient Questions Posted to a Public Social Media Forum | r/medicine | 33 |
